# Nanocellulose Coatings for Surgical Face Masks

**DOI:** 10.3390/nano16020112

**Published:** 2026-01-15

**Authors:** Divya Rajah, Sandya Athukoralalage, Ramanathan Yegappan, Nasim Amiralian

**Affiliations:** Australian Institute for Bioengineering and Nanotechnology (AIBN), The University of Queensland, Corner College and Cooper Roads, Brisbane, QLD 4072, Australia; divyarajah18@gmail.com (D.R.); s.athukoralalage@uq.edu.au (S.A.); r.yegappan@uq.edu.au (R.Y.)

**Keywords:** non-wood nanocellulose, sustainability, face masks, nonwoven, filtration materials

## Abstract

Polypropylene (PP) nonwovens are widely used as filtration layers in surgical face masks, but their hydrophobic, inert surfaces limit their ability to attach functional coatings that adjust pore size and improve mechanical filtration. Herein, we exploit cellulose derived from sugarcane debris to construct nanocellulose coatings that modify the surface properties of PP mask nonwovens without altering the underlying fibre architecture. Cellulose pulp was fibrillated to cellulose nanofibres (CNFs) and functionalised to yield TEMPO-oxidised nanofibres (TCNFs) and cationic nanofibres (CCNFs). All these nanofibres retain a cellulose I structure with a thermal stability of well above an 80–100 °C drying window. The three nanocelluloses exhibit distinct combinations of surface charge and wettability (ζ ≈ −9, −73, and +76 mV), with various hydrophobicity. Dip coating produces nanocellulose coating layers on PP, with uniform coverage at 1 wt% for TCNF and CCNF. CCNF inverts the negative surface charge of PP and maintains the positive charge at 86% relative humidity. Ethanol pretreatment of PP increases CCNF coating adhesion and preserves a continuous nanoporous CCNF film on the PP surface under humid conditions. Cytotoxicity assays indicate no detectable cytotoxicity for coated or uncoated nonwovens. This work establishes sugarcane-derived nanocellulose, particularly CCNF and TCNF, as a potential biocompatible surface coating for PP mask nonwovens.

## 1. Introduction

Disposable face masks and filtering facepiece respirators rely on multilayer polypropylene nonwovens, melt-blown and spunbond layers, to capture aerosol and particles [1]. Their performance and reliability are determined by the bulk fibre architecture of the melt-blown and spunbond layers, as well as the surface chemistry and electrostatic charge of the polypropylene (PP) fibres, which together control particle capture efficiency, breathability and fluid resistance. With increasing expectations for mask performance, comfort and safety, there is growing interest in functionalising established nonwoven substrates with thin coatings that (i) tailor surface wettability to improve moisture management and wearer comfort, (ii) strengthen interfacial interactions to enhance coating adhesion and durability, (iii) maintain functionality under humid, droplet-rich conditions encountered during use and (iv) potentially augment size-exclusion- based capture while preserving the underlying mechanical integrity and filtration architecture. However, the successful implementation of such coatings requires a detailed understanding of the coating’s assembly on complex nonwoven geometries, the strength and durability of their adhesion to inherently hydrophobic polymer fibres and their effects on cytocompatibility and other surface-mediated properties that are critical for prolonged contact with facial skin.

Nanocellulose (NC) offers an attractive platform for such functional coatings because it is derived from abundant cellulose resources, represents the fundamental building block of biomass and can be processed in water into colloidal dispersions that readily assemble into high-surface area networks on a wide variety of substrates [2]. Depending on the extraction route and morphology, NC is commonly classified into cellulose nanofibres (CNFs) and cellulose nanocrystals (CNCs). CNFs typically exhibit flexible, fibrillar morphologies with widths of tens of nanometres and micrometre-scale lengths, obtained by high-shear mechanical treatments such as high-pressure homogenisation [3,4,5]. CNCs, by contrast, are shorter, rod-like particles with higher crystallinity, typically 50–350 nm in length and 5–20 nm in width, produced using strong-acid treatments [5,6]. This tunability in size, aspect ratio and surface chemistry, combined with intrinsic biodegradability, mechanical robustness and ease of surface modification, has enabled the use of NC in applications ranging from textiles and water treatment to packaging and composites [2,7,8,9].

Non-wood cellulose resources derived from agro-industrial residues, such as sugarcane trash and bagasse, are particularly appealing as sustainable feedstocks, providing large volumes of underutilised cellulose from crop farming, food processing and horticulture [10,11]. For instance, sugarcane waste, consisting of trash and bagasse, constitutes a substantial agricultural residue globally available, attributed to the annual production of approximately 1.6 billion tons of sugarcane crops, yielding an estimated 279 million metric tons of waste annually [12]. In this context, cellulose fibres obtained from sugarcane trash and subsequently refined into NC fractions with distinct sizes and surface charges through combined chemical and mechanical treatments represent a promising basis for coatings on surgical face masks nonwoven to tailor surface morphology, charge and wettability while preserving the integrity of the micron-scale polypropylene fibre framework.

Despite growing interest in NC-based coatings for fibrous substrates, the behaviour of such coatings on commercial-type polypropylene (PP) nonwovens used in face masks, particularly their morphology at the nano scale, adhesion to hydrophobic substrates, wettability and cytotoxicity in mask-relevant conditions, remains insufficiently understood. In this study, we derive a series of NC fractions from sugarcane trash systematically varying their surface functionalities and apply them as ultrathin coatings to the middle layer material of surgical face masks. These results demonstrate the potential of integrating sustainable NC coatings derived from agro-waste into nonwoven mask media to modulate surface properties in a manner compatible with existing mask designs.

## 2. Result and Discussion

The washed and ground sugarcane was subjected to alkaline delignification, followed by bleaching, resulting in cellulose microfibre (CMF). CMF was subjected to mechanical shear forces by a high-pressure homogeniser (HPH) to produce cellulose nanofibres (CNFs). The cellulose backbone is rich in hydroxyl functionalities that serve as reactive sites for covalent modification, enabling the incorporation of cationic (quaternary ammonium) or anionic (carboxylate) groups. These surface charges not only impart electrostatic functionality but also promote fibrillation by enhancing fibre–fibre repulsion and reducing the formation of large aggregates.

Cationic cellulose was prepared by reacting cellulose pulp with 3-chloro-2-hydroxypropyltrimethylammonium chloride (CHPTAC) under alkaline conditions. In the presence of NaOH, CHPTAC is converted in situ into the reactive epoxide 2,3-epoxypropyltrimethylammonium chloride (EPTMAC), which subsequently undergoes nucleophilic ring-opening by deprotonated cellulose hydroxyl groups, leading to covalent attachment of quaternary ammonium functionalities along the cellulose chains [13,14]. The resulting cationised pulp is referred to as cationic cellulose microfibre (CCMF), and high-pressure homogenisation of CCMF produced cationic cellulose nanofibres (CCNFs). Anionic cellulose was obtained via TEMPO-mediated oxidation of cellulose pulp, a well-established route for introducing carboxylate groups at the C6 positions of the glucopyranose units [15]. In this reaction, TEMPO/NaBr/NaClO under mildly alkaline conditions (pH ≈ 10) selectively oxidises primary hydroxyl groups to carboxylate functionalities, as reflected by the consumption of NaOH required to maintain the reaction pH. The oxidised cellulose pulp is denoted TCMF, and subsequent mechanical homogenisation yielded TEMPO-oxidised cellulose nanofibres (TCNFs). These NC fibres (CNF, CCNF and TCNF) were investigated in the following sections, where we correlate their morphology and surface charge with coating behaviour on nonwoven PP substrates.

**Figure 1:**
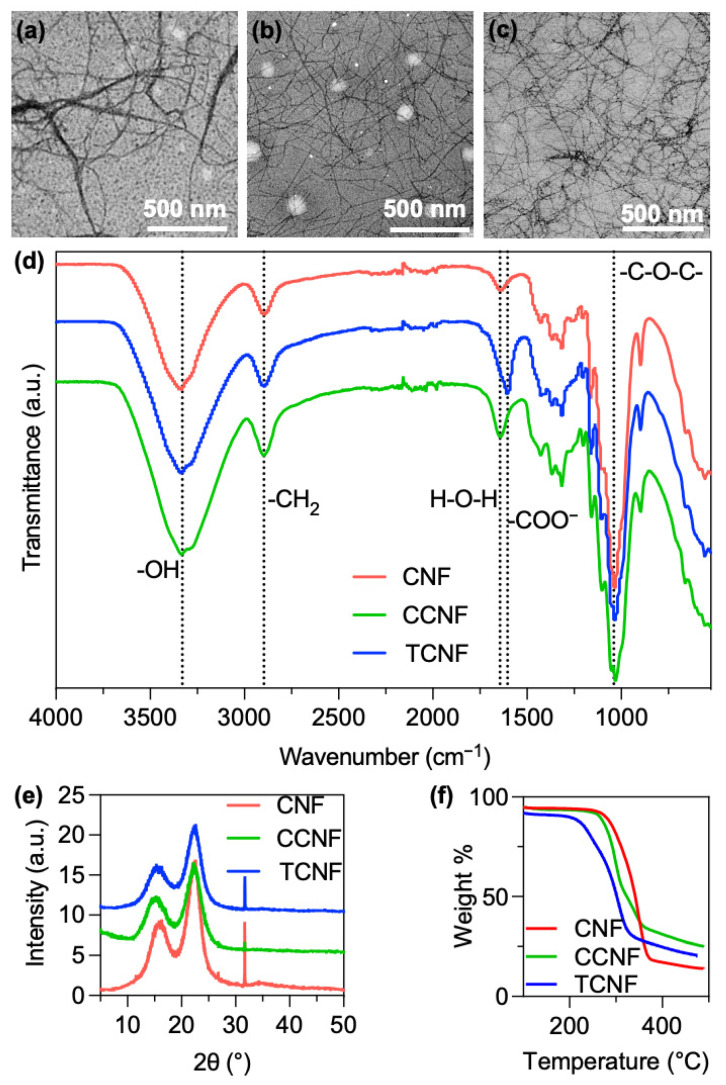
Characterisation of nanocellulose samples. TEM images of (**a**) CNF, (**b**) TCNF and (**c**) CCNF. (**d**) FTIR spectra, (**e**) XRD patterns and (**f**) TGA curves of CNF, TCNF and CCNF.

Zeta potential measurements in water confirmed the distinct surface chemistries of the three nanocellulose fractions. Unmodified CNF showed a low negative zeta potential of approximately −9 mV. Cationic CCNF exhibited a zeta potential of +76 mV with a degree of substitution of quaternary ammonium groups of ~10%, whereas TCNF displayed a zeta potential of −73 mV with a degree of oxidation of ~0.08 COO^−^ per anhydroglucose unit (8%). TEM analysis supported the charge-dependent dispersion behaviour (Figure 1). The unmodified cellulose material appeared as dense, multi-fibril bundles with a limited number of individual fibrils (Figure 1a), whereas both TCNF (Figure 1b) and CCNF (Figure 1b) formed long, flexible individual nanofibres with diameters below 10 nm and lengths of several micrometres. In the charged samples, only occasional thin bundles were observed, and the overall network was more open and homogeneous, consistent with the suppression of re-aggregation during and after homogenisation. The combination of high aspect ratio, sub-10 nm diameters and good individualisation in CCNF and TCNF provides a favourable starting point for forming thin, continuous coatings on PP nonwoven fibres, in contrast to the thicker, more heterogeneous deposits expected from bundle-dominated dispersions.

The homogenisation process of CMF to CNF is presumed to be solely a physical process rather than chemical alteration; hence, the chemical characterisation remains consistent across both micro- and nanofibres. FTIR spectroscopy (Figure 1d) was performed to confirm the functionalisation. In CNF, a broad band at 3377 cm^−1^ is assigned to the O-H stretching of cellulose, and the peak at 2897 cm^−1^ to the C-H stretching of methylene groups. The band at 1644 cm^−1^ corresponds to the H-O-H bending of absorbed water. Features in the 1426–1313 cm^−1^ region are attributed to CH_2_/CH_3_ deformation (bending) modes of the -N^+^(CH_3_)_3_ substituent introduced by CHPTAC [16]. The band at 1040 cm^−1^ is consistent with C-O-C (ether) stretching of the grafted cellulose-O-CH_2_-CHOH-CH_2_-N^+^(CH_3_)_3_ linkage [17]. For TCNF, the COO^−^ group from TEMPO oxidation can be identified at 1604 cm^−1^, which is indicative of successful carboxylate functionalisation.

XRD patterns of CNF, CCNF and TCNF (Figure 1e) showed reflections at 2θ ~18.6° and 22.4°, characteristic of the (110) and (200) planes of cellulose I, confirming that the native cellulose I structure is preserved in all nanofibres. The crystallinity index was ~70% for CNF and ~65% for CCNF, indicating that cationisation and subsequent homogenisation only slightly reduce the overall crystallinity. Similar values were obtained for TCNF, consistent with TEMPO oxidation and fibrillation mainly affecting the fibre surface rather than disrupting the crystalline structure.

Thermogravimetric analysis (TGA, Figure 1f) was used to assess the effect of surface modification on thermal stability. Unmodified CNF showed an onset of thermal degradation at ~277 °C, well above the intended drying temperature range. After cationisation, the onset temperature decreased to ~260 °C for CCNF, while TCNF exhibited a lower onset of ~217 °C, consistent with the introduction of quaternary ammonium and carboxylate groups that are less thermally stable than the native cellulose backbone. Such reductions in onset temperature have been attributed to the perturbation of intermolecular interactions and crystal packing in cellulose upon incorporation of charged functional groups [18]. Even with the reduction, all nanofibres remain thermally robust under the intended 80–100 °C nanocellulose coating drying conditions.

**Figure 2:**
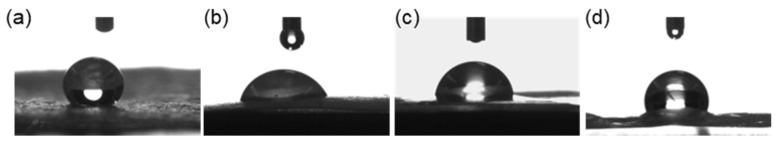
Contact angle measurements illustrate the wettability of various cellulose thin films: (**a**) PP nonwoven. (**b**) CNF, (**c**) TCNF and (**d**) CCNF films.

Static water contact angles were first measured on the uncoated PP nonwoven, which showed a contact angle of 128° (Figure 2a), confirming its hydrophobic character. To compare this with NC, contact angles were then measured on films prepared by vacuum filtration of CNF, CCNF and TCNF suspensions followed by drying. Films were used instead of coated nonwovens because the thin, porous coatings on PP did not allow reproducible contact angle measurements. CNF film showed a contact angle of 77° (Figure 2b), indicating a moderately hydrophilic surface. TCNF gave an intermediate value of 88° (Figure 2c), whereas CCNF films were more hydrophobic, with a contact angle of 107° (Figure 2d). The higher contact angle of CCNF is consistent with the introduction of quaternary ammonium groups bearing methyl substituents, which reduce surface polarity, while the carboxylate groups in TCNF maintain a more polar surface. Among the nanofibre samples, CCNF combines a strong positive surface charge (ζ = +76 mV) with the highest contact angle value; therefore, it was selected as the primary candidate for coating PP nonwoven substrates, with CNF and TCNF retained as comparative cases.

### 2.1. Evaluating Cellulose as a Coating Material

Dip coating was used to deposit nanocellulose coatings from aqueous dispersions, with a 20 s immersion followed by controlled withdrawal and drying. Initial trials with microfibre dispersions (CMF, CCMF, TCMF) produced thick, patchy coatings with uneven fibre coverage; therefore, they were not pursued further. Subsequent work focused on nanofibres, which gave markedly more homogeneous coatings.

**Figure 3:**
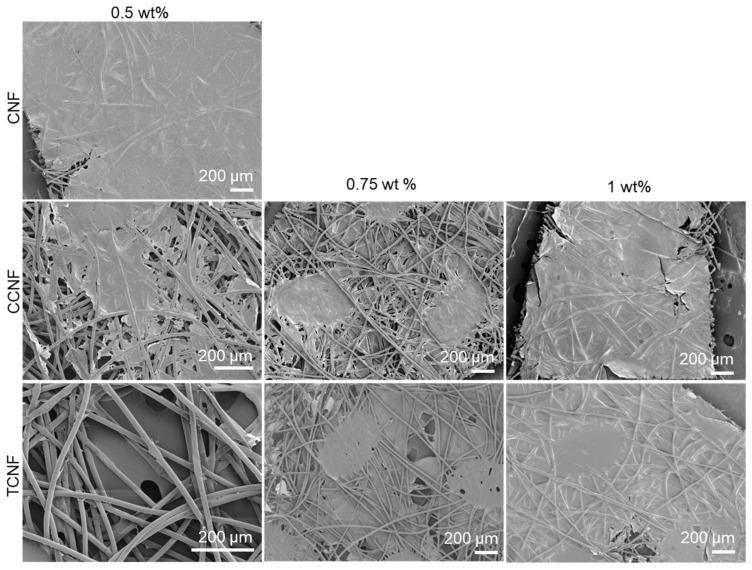
SEM images of nanofibre coatings (CNF, CCNF and TCNF) at various concentrations.

PP nonwovens were dipped into 0.5, 0.75 and 1.0 wt% dispersions of CNF, TCNF and CCNF. SEM images (Figure 3) show that all three cellulose nanofibres can form continuous coatings on the PP fibres, but the concentration required for full coverage depends on surface chemistry. At 0.5 wt%, CNF produced a largely continuous network on the fibres, with only small uncoated regions. TCNF and CCNF at the same concentration did not fully cover the PP fibres with large areas of uncoated fibres. Increasing the concentration to 0.75 wt% reduced the extent of uncovered fibres for TCNF and CCNF, and at 1.0 wt% both samples formed continuous coatings comparable in coverage to those obtained with 0.5 wt% CNF.

### 2.2. Zeta Potential Analysis

In dispersion, CNF, TCNF and CCNF carry distinct surface charges of ζ ~−9, −73 and +76 mV, respectively. To determine how these coatings modify the surface potential of the PP nonwoven and whether the charge is retained under use-relevant humidity, solid-state zeta potential measurements were performed on coated and uncoated substrates after equilibration at 65% relative humidity (RH) and 86% RH for 4 h to mimic the high humidity generated during breathing.

The uncoated PP nonwoven charge was strongly negative, with zeta potentials of −93 mV at 65% RH and −77 mV at 86% RH. Coating with CCNF reversed the sign of the surface potential and produced strongly positive values of +89 mV at 65% RH and +80 mV at 86% RH. TCNF-coated nonwovens remained negatively charged but with reduced magnitude compared to the uncoated substrate, showing −49 mV at both humidity levels. The zeta potential value of the CNF-coated samples, however, could not be quantified reliably. The coating partially detached during the aqueous measurement, due to the weaker adhesion bonding between with the nonwoven.

### 2.3. Adhesion of Nanocellulose Coatings to PP Nonwovens

**Figure 4:**
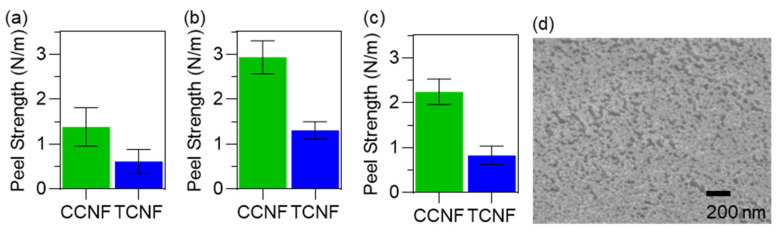
Adhesion of nanocellulose coatings on PP nonwovens measured by T-peel test. (**a**) Peel strength of CCNF and TCNF coatings on untreated PP. (**b**) Peel strength on ethanol-pretreated PP. (**c**) Peel strength on ethanol-pretreated PP after conditioning at 85 ± 5% RH. (**d**) SEM image of a 1 wt% CCNF coating on ethanol-pretreated PP nonwoven.

The adhesion strength between NC coatings and PP nonwovens was quantified by a T-peel test (ASTM D1876-08) [19]. Two 30 × 160 mm PP strips were coated with 1 wt% dispersion of CCNF or TCNF, pressed together, and dried at 85 °C for 4 h before testing. The strips were then peeled apart at 254 mm min^−1^, and the steady-state force was used to calculate the peel strength. On untreated PP, CCNF coatings gave an average peel strength of 1.29 N m^−1^, whereas TCNF coatings reached 0.69 N m^−1^ (Figure 4a).

To improve wetting and interfacial contact, the PP nonwoven was treated with ethanol prior to coating [20]. This treatment increased the peel strength of CCNF coatings to 2.93 N m^−1^, representing an increase of ~56% relative to untreated PP. For TCNF, the peel strength rose to 1.32 N m^−1^, an increase of ~53% (Figure 4b). The higher adhesion on ethanol-treated substrates is consistent with decreasing the surface tension and improved wetting of the PP fibres by the aqueous NC dispersions, which increases the real contact area and promotes stronger interfacial interactions. Adhesion was then assessed under humid conditions representative of mask use. Ethanol-pretreated, coated specimens were equilibrated at 85 ± 5% relative humidity in a KCl-based humidity chamber, following EN 14683:2025 [21], and tested immediately afterwards. Under these conditions, the peel strength of CCNF coatings decreased by ~24% compared to the dry ethanol-treated state, whereas TCNF coatings showed a larger reduction of ~37% (Figure 4c). The stronger humidity sensitivity of TCNF is attributed to the higher water affinity of carboxylate groups, leading to greater swelling of the nanocellulose layer and disruption of interfacial contacts [22]. In both cases, however, the peel strength after humidity exposure remained higher than those measured on coatings prepared on non-pretreated PP, indicating that ethanol pretreatment not only enhances initial adhesion but also improves adhesion retention under humid conditions.

Given the good adhesion of CCNF coatings on ethanol-pretreated PP, the coating morphology was examined. An SEM image of a 1 wt% CCNF coating on ethanol-pretreated PP (Figure 4d) shows a continuous surface layer with nanoscale features across the coated region. This indicates that the CCNF forms a coherent surface coating without evidence of dense film collapse, consistent with the intended use as a thin, surface-modifying layer on the nonwoven fibres.

### 2.4. Cytotoxicity of Modified Cellulose Coating

**Figure 5:**
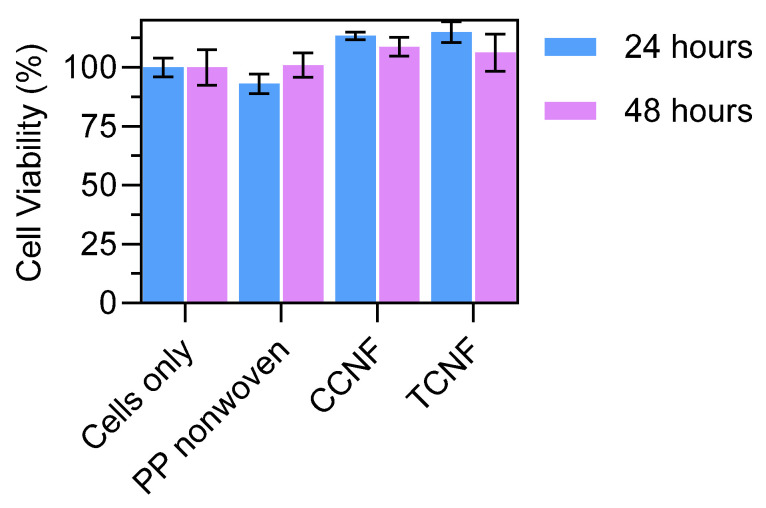
Cell viability of L929 fibroblasts exposed to extracts of uncoated PP nonwoven, CCNF-coated nonwoven and TCNF-coated nonwoven after 24 and 48 h.

The cytotoxicity of 1 wt% CCNF- and TCNF-coated nonwovens was evaluated using L929 murine fibroblasts in accordance with ISO 10993-5 [23]. Extracts of the uncoated PP nonwoven and of the coated samples were prepared in culture medium and incubated with L929 cells, and cell viability was quantified after 24 and 48 h. Materials are considered non-cytotoxic if cell viability exceeds 70% of the negative control (cells cultured in media only). Cell viabilities for extracts of the uncoated nonwoven, CCNF-coated nonwoven and TCNF-coated nonwoven were all well above the 70% threshold at both 24 and 48 h and were comparable to the control (Figure 5). No significant decrease in viability was observed for either CCNF or TCNF relative to the commercial PP nonwoven substrate. These results indicate that the modified cellulose coatings do not introduce measurable cytotoxic effects under the conditions tested and support the biocompatibility of CCNF- and TCNF-coated nonwovens for use in face mask applications.

## 3. Conclusions and Future Directions

We have shown that NC derived from sugarcane trash can be chemically tailored and integrated as thin coatings on PP nonwovens used in surgical face masks. CNF, TCNF and CCNF retained a cellulose I structure and sufficient thermal stability for 80–100 °C processing, while surface functionalisation produced distinct combinations of charge and wettability that govern coating behaviour. Dip coating yielded continuous layers on PP, with TCNF and CCNF achieving full coverage at 1.0 wt%. On the coated nonwovens, CCNF inverted the native negative surface potential of PP and maintained a strong positive zeta potential at 86% relative humidity, whereas TCNF reduced but did not reverse the negative potential. Ethanol pretreatment of the PP fibres substantially increased peel strength for both coatings and improved adhesion retention under humid conditions, and CCNF forms a continuous nanoporous film. Cytotoxicity assay showed no detectable cytotoxic response for any coated or uncoated substrates. These results establish sugarcane-derived NC, particularly CCNF and TCNF, as biocompatible, process-compatible, surface-active coatings for face masks. These results establish nanocellulose, particularly CCNF and TCNF, as biocompatible, process-compatible, surface-active coatings for polypropylene nonwovens. Future work will focus on optimising coating chemistry and thickness to meet the filtration efficiency and pressure drop standard requirements using established aerosol-based testing methodologies [24] reported for nanofibre and coated filter media.

## 4. Experimental Method

### 4.1. Materials

Sugarcane waste was obtained from Sunshine Sugar (Ballina, NSW, Australia). The reagents used were as follows: sodium hydroxide analytical reagent (NaOH: ChemSupply, Gillman, SA, Australia), glacial acetic acid (CH_3_COOH; Merck, Macquarie Park, NSW, Australia), sodium chlorite -Technical Grade 80% (NaClO_2_: Sigma-Aldrich, St. Louis, MO, USA), (3-chloro-2-hydroxypropyl) trimethylammonium chloride solution—60 wt% in H_2_O, (CHPTAC: Sigma-Aldrich, Castle Hill, Australia), hydrochloric acid, (HCl: Merck), silver nitrate ≥ 99% (AgNO_3_: Sigma-Aldrich), sodium bromide ≥ 99% (NaBr: Sigma-Aldrich), 2,2,6,6-tetramethyl-4-piperidin-1-yl)oxy (TEMPO: Sigma-Aldrich), sodium hypochlorite solution 6–14% *w*/*v* (NaClO: Merck) and sodium chloride (NaCl: Sigma-Aldrich), without further purification. The middle layer of the surgical face mask, 20 g per square metre (GSM) polypropylene nonwoven fabric, was provided by an industry, Evolve (Crestmead, QLD, Australia).

### 4.2. Pulping Sugarcane Trash

Pulping of sugarcane trash was carried out following a method previously described by Amiralian et al. [25]. Washed and dried sugarcane trash was ground into a fine powder using a cutting mill (SM 300, Retsch, Haan, NRW, Germany). A total of 100 g of this powder was dispersed in Milli-Q water at a 1:10 (*w*/*v*) ratio and stirred overnight at room temperature. The suspension was then delignified using a 2% (*w*/*v*) sodium hydroxide solution at 80 °C for 2 h, followed by rinsing with water at 60 °C. The delignified pulp was bleached with a 1% (*v*/*v*) sodium chlorite solution in a 1:30 pulp-to-water ratio, with the addition of glacial acetic acid (1:1 pulp-to-acid ratio, g/mL) and heated at 70 °C for 1 h. The bleaching process was repeated twice to remove residual lignin. The resulting white coloured cellulose pulp was thoroughly rinsed with 60 °C distilled water between each bleaching step.

### 4.3. CHPTAC Cationisation of Cellulose

Cationisation of bleached cellulose pulp was performed following Rol et al. with modifications [26]. A total of 50 mL of 10 M of NaOH solution was added dropwise to the 300 mL of 6.7 wt% of bleached pulp and the reaction was heated to 65 °C for 30 min followed by dropwise addition of 100 mL CHPTAC. The reaction mixture was heated up to 65 °C and stirred for 4 h, followed by neutralising to pH 7 with 0.1 M of HCl and centrifugation three times at 20,000 RPM for 10 min at 16 °C. The treated pulp was dialysed for 3 days to completely remove the unreacted CHPTAC and dissolved sugar.

The DS of CHPTAC functionalisation was determined using conductometric titration following the literature procedure [26]. A suspension of 0.1 wt% of the functionalised cellulose was titrated with 0.01 M AgNO_3_ aqueous solution by adding 200 µL in 60 s. The titration was repeated twice, and the average is reported. The DS was calculated following Equation (1) below:

(1) $$\mathrm{DS}=\frac{n_{{AgNO}_{3}}}{n_{cellulose}}=\frac{C_{{AgNO}_{3}}\;\times \;V_{{AgNO}_{3}}}{\frac{{mass}_{Cellulose}}{{MW}_{Cellulose\;}}}$$

where n_AgNO3_ (mol) is the number of mole of AgNO_3_ at the equivalence, n_cellulose_ (mol) is the number of mole of cellulose present in the beaker, C_AgNO3_ (mol/L) is the concentration of the solution of AgNO_3_, V_AgNO3_ (L) is the volume of AgNO_3_ used at the equivalence, mass_cellulose_ (g) is the quantity of cellulose added and MW_cellulose_ (g/mol) is the molar mass of cellulose.

### 4.4. TEMPO-Mediated Oxidation of Cellulose Pulp

The cellulose pulp was oxidised following the literature procedures [27]. The 1 wt% of cellulose pulp (5 g) was suspended in MilliQ water to which NaBr (0.51 g) and (2,2,6,6-Tetramethylpiperidin-1-yl)oxy (TEMPO, 0.08 g) were added. Sodium hypochlorite (NaClO, 156 mL) was adjusted to pH 10 using 1 M HCl and added to the suspended reaction mixture. The reaction mixture was stirred at room temperature for 4 h while maintaining the reaction mixture at pH 10 using 0.5 M NaOH. T-CMF was yielded via filtration and washing with MilliQ water.

The degree of oxidation (DO) of the carboxylic acid groups after TEMPO oxidation was determined via conductimetric titration following the literature procedure [28]. The carboxylate cellulose (0.55 wt%) and 0.01 M NaCl (5 mL) were stirred for 15 min followed by adjusting the pH of the suspension between pH 2.5 and pH 3 using 0.1 M HCl. The reaction mixture was titrated with 0.04 M NaOH. Titration was repeated twice. The DO was calculated following equation [7].

$$\mathrm{DO}=\frac{162\times C_{NaOH}\times {\left(V_{2-}V_{1}\right)}_{NaOH}}{w_{cellulose}-36\times C_{NaOH}\times {\left(V_{2}-V_{1}\right)}_{NaOH}}$$

where C_NaOH_ is the concentration of NaOH (mol L^−1^), V_1_ and V_2_ is the volume of NaOH (L) and w is the dry weight of cellulose used (g)

### 4.5. Mechanical Processing Using High-Pressure Homogenisation

Upon chemical treatments, the modified cellulose microfibres (CMF) were homogenised to produce cellulose nanofibres (CNF) using a high-pressure homogeniser (HPH) (PandaPLUS 2000, GEA Mechanical Equipment Italia S.p.A, Parma, Italy). A total of 0.1–0.5 wt% of microfibres were passed through the homogeniser, with one pass at 400 bar, one pass at 800 bar and three passes at 1100 bar.

### 4.6. Dip Coating of Nonwoven Fabric

In total, 0/5, 0.75 wt% and 1.0 wt% dispersions of CNF were prepared and poured into a rectangular glass tank for coating the 20 GSM polypropylene nonwoven fabric. The nonwoven was cut into 10 × 10 cm and clamped to the metal frame and immersed into the CNF dispersion. The fabric was gently shaken for a few seconds, ensuring even coverage on one side. After dipping for 20 s, the frame was slowly lifted out of the nanocellulose dispersion, allowing the excess solution to drain off, followed by hanging the coated fabric on a drying rack in an 80 °C convection oven for 45 min to dry completely. The coated nonwovens were either conditioned at room temperature or at 85% humidity using KCl salt (under face mask respiration) for 24 h to investigate the coating efficiency.

### 4.7. Fourier Transform Infrared (FTIR)

FTIR spectra were measured in transmittance mode using a Thermo Nicolet 5700 ATF-FTIR spectrometer (Thermo Fisher Scientific, Madison, WI, USA) with a diamond-attenuated total reflectance recorded from 525 to 4000 cm^−1^ wavenumbers. All samples were measured in solid-state after freeze-drying.

### 4.8. Thermogravimetric Analysis (TGA)

The thermal stability of the samples was investigated using Mettler Toledo TGA/DSC 1 Star (Mettler Toledo, Zurich, Switzerland). The freeze-dried samples were heated from 40 °C to 500 °C at a heating rate of 5 °C min^−1^ under nitrogen.

### 4.9. Transmission Electron Microscopy (TEM)

A 0.0005 wt% suspension of nanocellulose samples was sonicated for 5 min at 20% amplitude for 6 s on and 2 s off using a QSONICA Sonicator (Heat Systems Ultrasonics, Brookfield, CT, USA). A total of 10 µL was drop-casted on a carbon-coated grid and left to dry. The grid was negatively stained with 2% Uranyl acetate for 5 min and samples were analysed using HT7700 operating at 100 kV (Hitachi High-Technologies Corporation, Tokyo, Japan).

### 4.10. Scanning Electron Microscopy (SEM)

SEM imaging of the CNF-coated nonwoven was conducted using a JEOL JSM-7800F field emission scanning electron microscope (JEOL Ltd., Tokyo, Japan). Samples were sputter-coated with a 15 nm layer of platinum to enhance conductivity and were imaged at an accelerating voltage of 5.0 kV.

### 4.11. X-Ray Diffraction (XRD)

XRD analysis was performed using a Bruker D8 Advance diffractometer (Bruker, Karlsruhe, Germany) equipped with a 0.2 mm slit and graphite-filtered Cu-Kα radiation, operated at 30 kV and 20 mA. Samples were mounted on a sample holder and scanned over a 2θ range of 5° to 75° at a scanning rate of 1° per minute. The crystallinity index (CI) was calculated using the Segal method (Segal et al., 1959), as shown in the equation below [29]:

$$\mathrm{CI}\;=\;\frac{I_{200}\times I_{am}}{I_{200}}\times 100\%$$

where I_200_ represents the intensity of the peak at 2θ ≈ 22.4°, which is the main peak for the crystalline phase of cellulose and the intensity of the peak at 2θ ≈ 16°, I_am_, the valley between the crystalline peaks of cellulose polymorph Iβ 1–10 (15°), 110 (16.6°) and 200 (22.4°) planes represent the amorphous domain.

### 4.12. Contact Angle (CA)

CA was measured by DataPhysics Instruments OCA 14EC/B Contact Angle Measurement Instrument (DataPhysics Instruments GmbH, Filderstadt, Germany) and analysed using SCA202 software (version 6.1). A water droplet (5 µL) was dispensed using an automated pump and the inbuilt camera captured the images to measure the static contact angle using the ellipse fitting method. All measurements were conducted at room temperature and recorded at 60 s after the water droplet had deposited onto the surface of the cellulose thin film. The cellulose thin film was made from 40 mL of 1 wt% solution using vacuum filtration followed by drying at 110 °C for 20 min with 0.9 kPa vacuum pressure.

### 4.13. Zeta Potential

The treated CNFs were diluted to 0.005 wt% to measure the zeta potential via Zetasizer (ZSU5700 Ultra, Malvern Panalytical, Worcestershire, UK). The samples were placed in DTS1070 cells (Malvern Panalytical, Worcestershire, UK) and measured at room temperature.

### 4.14. Solid-State Zeta Potential

Surface charge of coated surfaces was measured with Zetasizer Ultra from ATA Scientific (Caringbah, NSW, Australia). It is worth acknowledging the shortcoming of the instrument, which is that the measurement values could be influenced by the size and length of the fibres. To investigate the charge of nanocellulose after drying and after conditioning at 85% humidity, the charge of the CNF-coated nonwoven was measured with Surpass Anton Parr using 0.1 M KCl solution at pH 5 using an adjustable gap cell. The measurement was repeated twice, and each measurement gives an average of 4 replicates. The zeta potential is calculated based on the derivative of the Helmholtz–Smoluchowski equation below:

$$\zeta \;=\;\frac{{dU}_{str}}{d∆p}\times \frac{\eta }{ԑ\times {ԑ}_{0}}\times \kappa_{B}$$

where dU_str_/d∆p is the slope of streaming potential vs. differential pressure, $\kappa_{B}$ is electrolyte conductivity, η is the electrolyte viscosity, ε is the dielectric coefficient of the electrolyte and ε_0_ is the permittivity.

### 4.15. T-Peel Test

To evaluate the adhesion between the CNF coating and the nonwoven substrate, a T-peel test was conducted in accordance with the ASTM D1876-08 standard. Nonwoven material was cut into strips measuring 30 mm by 160 mm and coated with a 1.0 wt% CCNF solution. The coated strips were then dried at 85 °C for 4 h. Adhesion strength was measured using an Instron testing machine, applying a peeling force of 5 N m^−1^ at a rate of 254 mm min^−1^. To assess the influence of humidity on adhesion performance, additional samples were conditioned at 80% relative humidity for 24 h using a saturated KCl solution prior to testing.

### 4.16. Cytotoxicity

L929 murine fibroblasts were cultured in Dulbecco’s Modified Eagle Medium (DMEM) supplemented with 10% Foetal Bovine Serum (FBS) and 1% Penicillin–Streptomycin (Pen–Strep) at a controlled environment of 37 °C and 5% CO_2_. Upon reaching 90% confluence, cells were detached using 1× TrypLE. The cells were seeded into 96-well plates at a density of 8000 cells per well in 100 µL of culture medium and maintained under standard culture conditions. Sample with 1 cm × 1 cm of 1 wt% C-CNF-coated nonwoven fabric and 1 wt% T-CNF-coated nonwoven fabric were immersed in Dulbecco’s Modified Eagle Medium (DMEM) at 37 °C overnight. After overnight incubation, cells in the well plate were treated with leach-outs of the samples in culture media and incubated at 37 °C with 5% CO_2_ for 24 and 48 h periods. A total of 100 µL 3-(4,5-dimethylthiazol-2-yl)-2,5 diphenyltetrazolium bromide (MTT) solution was added to the cells, and the plates were incubated for 3 h. Following incubation, the intracellular formazan crystals were dissolved by the addition of 150 µL acidified isopropanol. The absorbance of the resulting suspension was measured at 590 nm, and cytotoxicity results were expressed as the percentage of viable cells cultured.

## Data Availability

The original contributions presented in this study are included in the article. Further inquiries can be directed to the corresponding author.
